# Evaluation of Resettin® on serum hormone levels in sedentary males

**DOI:** 10.1186/s12970-014-0043-x

**Published:** 2014-08-23

**Authors:** Mark L Anderson

**Affiliations:** 1 R&D Department, Triarco Industries, Wayne, NJ, USA

**Keywords:** Testosterone, Estradiol, Dihydrotestosterone, 5-alpha-reductase, Aromatase inhibitor, Resistance training, Sarcopenia

## Abstract

**Background:**

Comparisons of hormones such as dihydrotestosterone (DHT), estradiol (E2), and testosterone indicate their impact on metabolism and body composition. While less is known regarding DHT and E2, testosterone is an androgenic metabolic hormone capable of positively regulating a variety of anabolic and androgenic processes in the body. Accordingly, it has been postulated that the age-related reduction in serum testosterone levels leads to reductions in lean muscle mass, bone mineral density, and other physical conditions that impair physical performance and decrease quality of life. Preliminary studies suggest that key ingredients found in Resettin®/MyTosterone™, a natural supplement containing the carotenoid astaxanthin from *Haematococcus pluvialis* and Saw Palmetto berry lipid extract from *Serenoa repens*, could positively impact testosterone levels. To investigate the clinical efficacy of Resettin®, the serum profiles of testosterone, E2 and DHT in healthy sedentary males before and after Resettin® treatment were evaluated in a randomized, placebo controlled clinical trial.

**Method:**

Twenty healthy, sedentary men between the ages of 21 and 70 were randomized into either an 800 mg/day or 1200 mg/day Resettin®/MyTosterone™ treatment group or lecithin, which was used as the placebo. After a 14-day treatment period, there was a 14-day washout period. After the wash-out period, participants were crossed over within their respective group to either Resettin®/MyTosterone™ or the lecithin placebo for 14 days.

**Results:**

After 14 days, participants receiving 800 mg per day of Resettin® had significantly reduced baseline-subtracted serum DHT levels in comparison to the placebo control group. While after 14 days, participants receiving 1200 mg per day of Resettin® had significantly reduced baseline-subtracted serum DHT and E2 levels in comparison to the placebo control group. Moreover, participants receiving 1200 mg per day of Resettin® experienced a 38% increase in serum testosterone levels in comparison to the placebo control group, but the effect did not reach statistical significance.

**Conclusion:**

Although additional studies will be required to evaluate how Resettin® may promote proper testosterone regulation, these findings indicate that Resettin® can favorably influence serum hormone profiles in men.

## Background

Aging is associated with a decline in a variety of endocrine functions including menopause in women and a deterioration in androgen production in men [1]. Gradual reductions in testosterone levels can lead to many symptoms of andropause including a lack of energy, decreased mental acuity, a loss of overall well-being, and sexual dysfunction [2]–[4]. Androgen deficiency in aging men may also occur concomitantly with a geriatric syndrome called sarcopenia or the loss of significant amounts of lean skeletal muscle mass [5]. Sarcopenia is significantly associated with a variety of adverse outcomes which can result in increased incidences of slips, trips and falls leading to bone fractures, hospitalization and physical disability leading to a poor quality of life [6].

Although the causal factors leading to sarcopenia are complex and multifactorial, there is a clear association between age-related decreases in testosterone levels and increased incidences of sarcopenia [2],[6]. In males, testosterone is predominantly synthesized by Leydig cells of the testes using the steroid biosynthesis pathway. Testosterone acts on target cells expressing the androgen receptor to induce changes in gene expression related to the anabolic growth of muscle and an increase bone density, as well as the androgenic maturation of sex organs. Testosterone levels are directly regulated by 5α-reductase, an enzyme which catalyzes and regulates the synthesis of the more potent androgenic steroid hormone dihydrotestosterone (DHT) from free testosterone, and aromatase, an enzyme that directly converts testosterone into the estrogenic steroid hormone estradiol [7].

As men age, bioavailable levels of testosterone decrease by 2% per year after age 30 [8]. Given the role of testosterone in directly increasing the synthesis of muscle protein and counteracting the catabolic effects of the hormone cortisol in breaking down muscle, researchers and clinicians have developed a variety of pharmacological treatment modalities that aim to increase serum testosterone levels. These approaches include the exogenous administration of testosterone transdermally, through subcutaneous or intramuscular injections, or by boosting endogenous testosterone through the pharmacological inhibition of 5α-reductase inhibitors [9]. The latter approach is not a common clinical strategy as inhibitory drugs only elicit a moderate impact on testosterone (approximately 15%) in conjunction with an increase in E2, gynecomastia, erectile dysfunction, cataract formation, depressive symptoms, and other mood disorders [4],[10]–[14].

Currently, the most common approach for elevating testosterone levels is through the use of selective estrogen receptor modulators (SERMs), human chorionic gonadotropin (HCG), or a combination of both. SERMs block the effects of estrogen in the central nervous system and breast in men, thereby reducing the occurrence of gynecomastia and they also block the suppressive effect of estrogens on luteinizing hormone production, which propagates testosterone production [15]. HCG is structurally similar to the luteinizing hormone and it is recognized by the body as luteinizing hormone, which in turns signals the testes to begin producing more testosterone. However, SERMs also function as estrogen agonists in the liver and this leads to an increase in the production of the sex hormone binding globulin (SHBG), which circulates in the blood and may irreversibly bind to testosterone and other sex hormones, causing them to become inactive. As a result, SERMs therapy may increase the total concentration of testosterone, but the concentration of bioactive testosterone may remain low [15]. Furthermore, testosterone therapy has the potential to disrupt the feedback cycle from the hypothalamus/pituitary to the testes [16].

With regard to CVD it is uncertain that any risk or beneficial effects of increasing testosterone levels through exogenous testosterone therapy, SERMS or HCG may be different than the use of other approaches such as the use of natural supplements and is continuously under investigation. One such natural compound is Astaxanthin (AX), a carotenoid with favorable pharmacokinetics and bioavailability produced by *Haematococcus* algae (*pluvialis*) [17]. AX is shown to inhibit both 5α-reductase and aromatase CYP-19, which is an enzyme that converts C19 androgens to aromatic C18 estrogenic steroids [18],[19]. Moreover, findings from an open label dose response study of a product containing AX provided some suggestion that the compound may be involved in the regulation DHT and E2 levels, even within three days of treatment [19]. Thus, the primary aim of this study was to extend these findings to men under the age of 50. To this end, the hormonal response patterns of sedentary men was tested following an administration of novel Resettin®/MyTosterone™, which is a raw material consisting of AX and a lipid extract from the saw palmetto berry.

## Methods

### Study design

A prospective single blind treatment vs. placebo study was conducted over a 14 day period at Hunter Laboratories in Walnut Creek, CA. Experimental groups of 20 healthy, sedentary men between the ages of 21 and 70 years of age with a body mass index of less than 27 kg/m2 were phlebotomized following an overnight fast. Assignment to an experimental group was conducted in an alternating fashion, based upon arrival time. The study consisted of two experimental groups. In the low dose group, participants received a dose of 800 mg/day. The high dose group received a dose of 1200 mg/day. Study participants were asked to self-administer two to three (depending on their experimental group) soft-gelatin capsules daily containing either 400 mg of Resettin® (Resettin®/MyTosterone™; Triarco Industries, Wayne, NJ) or lecithin, which was used as the placebo. Participants were randomized into either the 800 mg/day or 1200 mg/day Resettin®/MyTosterone™ treatment group. After a 14-day treatment period, participants discontinued placebo or Resettin®/MyTosterone™ treatment for a consecutive 14 days. Following this 14-day washout period, participants were crossed over within their respective group to either Resettin®/MyTosterone™ or the lecithin placebo for 14 days. Blood was collected on days 0, 3, 7 and 14 days following the initiation of treatment. Serum hormone levels were collected and analyzed. Patterns of hormonal response were compared across the treatment groups in a pairwise manner. Researchers attempted to collect blood samples from all of the participants at approximately the same time of day in order to minimize circadian variations in serum hormone levels.

### Participants

A total of forty sedentary, healthy men between the ages of 21 and 68 met inclusion criteria and were enrolled for this study. Enrollment was voluntary, and participants signed informed consent statements in compliance with the Human Subjects Guidelines of Western Institutional Review Board and the American College of Sports Medicine. Participants were excluded from study if they had a history of smoking, pulmonary disease, hypertension, hepatorenal disease, musculoskeletal disorders, neuromuscular or neurological diseases, autoimmune diseases, cancer, peptic ulcers, or anemia. Participants were also excluded if they exhibited repeated signs of benign prostate hypertrophy, regularly consumed commercially available products containing saw palmetto or AX, were taking ergogenic levels of nutritional supplements that may affect muscle mass, such as creatine, or exhibited anabolic hormone levels, such as androstenedione or dehydroepiandrosterone. Participants taking prescription medication for a heart condition, pulmonary or thyroid problem were also excluded from the study. Participants on anti-hyperlipidemia, hypoglycemic, anti-hypertensive, endocrinologic, psychotropic, neuromuscular/neurological, or androgenic medications were also not invited to enroll in the study. After a 10-hour fast of all food or drink with caloric value along with a 48-hour rest from strenuous exercise, participants were phlebotomized. Participant age, height, weight, body mass index, resting heart rate, and blood pressure were also collected at this time.

### Serum hormone quantification

Serum levels of testosterone, DHT, and E2 were determined by enzyme-linked immunosorbent assay (ELISA) using commercially available kits (Alpha Diagnostic, San Antonio, USA). Briefly, reference controls, standards and samples were aliquoted in triplicate into separate wells pre-incubated with horseradish peroxidase (HRP)-conjugated primary antibodies specific for either testosterone, DHT, or E2. After washing, reference controls, standards, and sample wells were incubated with tetramethylbenzidine and gently agitated. After 10 min, the HRP-substrate colorimetric reaction was stopped with 0.16 M H2SO4, and the absorbance at 450 nm of each well was evaluated using a spectrophotometer.

### Statistical analysis

To evaluate the significance of possible group differences in steroid hormone levels within treatment groups, a 2 (high versus low dose) × 4 (sample time point) one-way repeated measures Analysis of Variance (ANOVA) was conducted. To evaluate statistically significant differences in steroid hormone levels between treatment groups, a two-way ANOVA was used. Differences in steroid hormone concentrations were considered clinically significant when the probability of a Type I error was less than 0.05.

## Results and discussion

Total testosterone levels tend to decline as men age [7]. Given that natural 5α-reductase/aromatase inhibitors, such as AX, may increase serum testosterone [9],[18],[19], we set out to determine if Resettin® was capable of increasing serum testosterone levels in sedentary men. To that end, a randomized controlled clinical dose comparison study of Resettin® was completed.

### Body weight, blood pressure, and tolerance

The average baseline body weight of participants within the 800 mg/day placebo and Resettin®/MyTosterone™ treatment groups were 88.3 kg and 93 kg, respectively. The average baseline body weight of participants within 1200 mg/day placebo and Resettin®/MyTosterone™ treatment groups were 103.7 kg and 95.8 kg, respectively. Results indicated that there were no clinically significant changes in average body weight over the duration of the study.

The average baseline systolic diastolic blood pressure ratios were 120 mmHg over 82 mmHg in the 800 mg/day placebo control group, 125 mmHg over 83 mmHg in the 800 mg/day Resettin®/MyTosterone™ treatment group, 122 mmHg over 82 mmHg in the 1200 mg/day placebo control group and 122 mmHg over 81 mmHg in the 1200 mg/day Resettin®/MyTosterone™ treatment group. No significant changes in systolic or diastolic blood pressure were observed over the course of the study. Owing to the significant safety profile and tolerability of AX as well as the other constituent compounds of Resettin®, there were no reports of adverse side effects during the study.

### Serum testosterone, E2, and DHT levels after Resettin®/MyTosterone™ treatment

**Figure 1:**
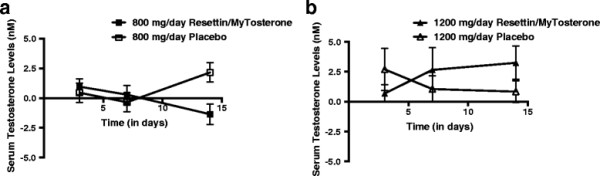
**Baseline subtracted serum testosterone levels in placebo- and Resettin®/MyTosterone™-treated participants.** Shown are the total serum testosterone levels from participants after 3, 7 and 14 days of 800 mg/day placebo **(a)** or Resettin®/MyTosterone™, or 1200 mg/day placebo or Resettin®/MyTosterone™ **(b)** as determined by ELISA. Each experimental group had between 9 and 10 participants, and results are indicative of one trial. Error bars denote standard deviation of the experimental mean.

To test for changes in serum testosterone level in response to Resettin®/MyTosterone™, participants were asked to self-administer orally either 800 mg/day or 1200 mg/day of Resettin®/MyTosterone™. In terms of the 1200 mg/day experimental group, the average serum testosterone levels were higher following 14 days as compared to the levels measured at baseline (day 0). For the 800 mg/day Resettin®/MyTosterone™ treatment group, the level of serum testosterone did not differ significantly between baseline and following 14 consecutive days of treatment (ANOVA-RM; p > 0.05). Serum testosterone levels for both groups are illustrated graphically in Figure 1. Furthermore, the results indicated that the serum testosterone levels of participants who were administered 1200 mg/day of Resettin®/MyTosterone™ were 38.04% higher than the serum testosterone levels of participants in the placebo control group Figure 1. However, there were no statistically significant differences in the average serum testosterone levels of either the 800 mg/day or 1200 mg/day Resettin®/MyTosterone™ treatment groups when compared to participants within the respective placebo control groups (ANOVA-RM; p > 0.05).

**Figure 2:**
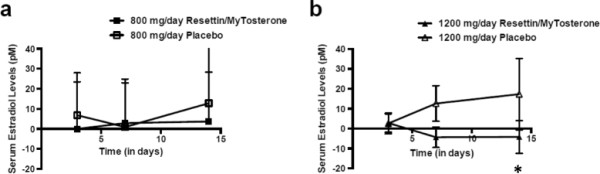
**Baseline subtracted serum E2 levels in placebo- and Resettin®/MyTosterone™-treated participants.** Shown are the serum E2 levels from participants after 3, 7 and 14 days of 800 mg/day placebo or Resettin®/MyTosterone™ **(a)**, or 1200 mg/day placebo or Resettin®/MyTosterone™ **(b)** as determined by ELISA. Each experimental group had between 9 and 10 participants, and results are indicative of one trial. There was a statistically significant reduction in the average serum E2 concentration of the 1200 mg/day Resettin®/MyTosterone™ treatment group compared to that of the 1200 mg/day placebo control group (ANOVA-2; p < 0.05). Error bars denote standard deviation of the experimental mean. An asterisk (*) indicates statistical significance.

Given that aromatase is capable of converting testosterone into E2, the serum concentrations of E2 were also evaluated by ELISA in all participants. Serum E2 levels did not significantly change relative to baseline levels. Further, there were no significant differences in the average serum E2 levels of the participants in the 800 mg/day and 1200 mg/day Resettin®/MyTosterone™ treatment groups as compared to the placebo control groups (Figure 2; ANOVA-RM; p > 0.05). Interestingly, when all serum E2 concentrations were adjusted by subtracting their baseline concentrations, results revealed a statistically significant reduction in the average serum E2 concentration of the 1200 mg/day Resettin®/MyTosterone™ treatment group compared to that of the 1200 mg/day placebo control group (Figure 2; ANOVA-2; p < 0.05).

**Figure 3:**
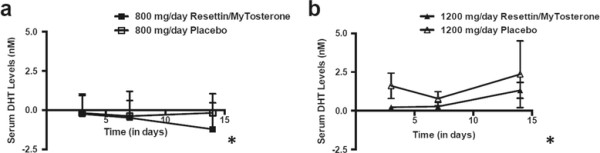
**Baseline subtracted serum DHT levels in placebo- and Resettin®/MyTosterone™-treated participants.** Shown are the serum DHT levels from participants after 3, 7 and 14 days of 800 mg/day placebo or Resettin®/MyTosterone™ **(a)**, or 1200 mg/day placebo or Resettin®/MyTosterone™ **(b)** as determined by ELISA. Each experimental group had between 9 and 10 participants, and results are indicative of one trial. There was a statistically significant decrease in the DHT levels in the 800 mg/day and 1200 mg/day Resettin®/MyTosterone™ treatment group compared to their respective placebo control groups (ANOVA-RM; p < 0.05). Error bars denote standard deviation of the experimental mean. An asterisk (*) indicates statistical significance.

The increase in serum testosterone levels for the 1200 mg per day of Resettin®/MyTosterone™ treatment group after 14 days was not statistically significant in comparison to the placebo group. However, there was a statistically significant decrease in the DHT levels in the 800 mg/day and 1200 mg/day Resettin®/MyTosterone™ treatment groups compared to their respective placebo control groups (Figure 3; ANOVA-RM; p < 0.05). Consistent with this data were the baseline-subtracted serum DHT levels in the 1200 mg/day Resettin®/MyTosterone™ treatment group which significantly decreased when compared to the serum DHT levels of the 1200 mg/day placebo control group (Figure 3; ANOVA-2; p < 0.05). These findings suggest that Resettin®/MyTosterone™ at the tested concentrations (800 mg/day and 1200 mg/day) do not significantly impact the serum levels of testosterone in sedentary men, but may have an impact on reducing serum E2 and DHT levels, which may in turn prevent the further reduction of testosterone levels.

## Conclusions

Deficiencies in testosterone production and the deregulation of testosterone’s anabolic activities are hallmarks of an aging endocrine system [1]. It is well-established that decreases in testosterone level are associated with a variety of medical problems, including a decline in cognitive function, loss of libido, loss of lean muscle mass and strength, and reductions in bone mineral density [2]–[4]. While the administration of exogenous testosterone can greatly ameliorate the deleterious effects of a testosterone deficiency, adverse side effects such as an imbalance in the hypothalamic-pituitary axis associated with this type of treatment option [16],[20]. By naturally increasing endogenous testosterone levels, the goal is to target the human body’s own well-regulated hypothalamic-pituitary-gonadal axis, whose function is to maintain homeostasis.

Published research suggests that supplementing AX may have inhibitory effects on 5α-reductase, thus, the primary aim of the current study was to evaluate the effects of the natural supplement, called Resettin®, on serum testosterone levels [18],[19]. Specifically, it was hypothesized that individuals who undergo treatment with Resettin® would have significantly higher serum levels of testosterone than those receiving the placebo. As illustrated in Figure 1, there were no statistically significant changes in serum testosterone levels following 14 days of treatment.

These findings are somewhat surprising, as they are in contrast to similar existing studies within the literature that demonstrated an elevation of testosterone after therapeutic treatment [9],[15]. Specifically, a number of previous studies have indeed found significant increases in serum testosterone levels within populations of men. Differences in terms of the participant population may account for why the present findings failed to support that of the extant literature. Specifically, there were meaningful differences in terms of the mean participant age across studies (i.e., 55.6 versus 41.2 years of age). Thus, age-related changes likely explain the lack of significant findings, as it is expected that the way that the body metabolizes, or processes, various supplements will produce variable results within and between populations. Changes related to typical aging are also likely have significant impacts on all processes within the body, and the synthesis of testosterone is no different. Moreover, other differences in sample population characteristics likely account for the divergent findings across these studies.

More specifically, compared to a non-placebo controlled trial conducted by Angwafor and Anderson [19], the present sample had many unique characteristics, which may be meaningful in terms of the generalizability of these data. For example, mean baseline concentrations of serum testosterone across the groups were measured to be less than half of the observed concentration levels at baseline in the previous study. Initial observation of DHT concentration across the groups were almost three times higher in the present study than the baseline DHT serum concentrations observed across all groups in the previous study. Baseline concentrations of estradiol between the two studies were even more divergent. Serum concentrations of estradiol at baseline across the groups were nearly four times higher in the current sample than that of the baseline serum estradiol concentrations observed previously. This is suggestive of underlying sample population characteristics that may account for variable results and additional studies exploring for latent clinical profiles of the androgen response to supplements are needed.

Indeed, participants in the present study weighed 10 kg to 15 kg more than the participants in the 2008 non-placebo controlled trial [19]. A wealth of evidence exists linking the accumulation of adipose tissue with detrimental metabolic changes within the body [21]–[24]. For instance, the aromatase enzyme, which is found within adipose tissue, is primarily responsible for converting androgens into estrogen. Increases in adipose tissue have been linked with higher serum concentrations of estrogens and lower levels of serum testosterone [21],[23]. As previously discussed, the men within the present sample exhibited much higher serum estrogen concentrations than the men in the previous study. Taken together, it is likely that metabolic changes as a result of being overweight or obese transform the manner in which the endocrine system is influenced through exogenous factors, such as dietary supplements.

In comparing serum estrogen concentration, responses to Resettin®/MyTosterone™ were different across both studies. Following baseline subtraction, average serum estrogen concentrations for an individual in the aforementioned study [19] were found to decrease significantly from baseline to day 7 in the low dosage group (800 mg/day), as well as from baseline to days 3, 7, and 14 in the high dosage group (2000 mg/day). Interestingly, the present study found similar patterns with a much lower dose of the supplement such that serum estrogen concentrations were found to be lower on average for the high dosage treatment group (1200 mg/day). The placebo group, in contrast, exhibited higher concentrations of estrogen overall. These data also support the idea that the metabolic profiles of participants in the current sample may not be comparable to that of the previous study, owing to confounding factors related to higher amounts of adipose tissue. Indeed, according to recently published data, estrogen levels for adult males typically range from between 37 to 110 pM [25]. Baseline concentration levels of participants in the current study ranged from 85 to 90 pM, while they ranged from 21.5 to 24 pM in the previous study. In conjunction, serum DHT concentrations were much higher at baseline in the present sample compared to the previous study. Interestingly, despite these differences, at day 14 the groups in both studies exhibited lower concentrations of serum DHT when compared to the placebo group. More specifically, in the current study the low dose group (800 mg/day) started out with concentrations of 6 nM of serum DHT and dropped more than 0.6 nM over the course of 14 days. Further, the high dosage group (1200 mg/day) exhibited an increase in serum DHT concentrations to approximately 1 nM at day 14, while the DHT levels for the placebo group also rose to approximately 2 nM. These data indicate that, given the likely contribution of higher levels of adipose tissue among participants in the present sample, it may be beneficial to examine the endocrine response, particularly testosterone levels, using a higher dose of Resettin®/MyTosterone™. Further, individuals included in the present sample were drawn from the U.S. population, while participants from the previous study were drawn from a country in west Central Africa. Thus, it is expected that factors related to the diet of individuals within the present sample, characterized as high in fats, sugars, and carbohydrates, as well as physical conditions, play a significant role in accounting for why the current data failed to replicate previous findings. Additional studies are warranted to provide support for the generalizability of these findings.

Further, the sample sizes across studies are relatively small [19]. Thus, there is a high risk for confounding factors that may have skewed the data. For instance, an unmeasured characteristic of the men included within the present study like higher levels of the aromatase enzyme, may account for their lack of response to Resettin®. Additional studies are warranted to more clearly delineate the association between Resettin® and serum testosterone levels. Findings from these studies are expected to improve the generalization of the conclusions. Notwithstanding, there was a measurable 38% increase in serum testosterone levels and a 4.5% decrease in estradiol among participants receiving the 1200 mg/day experimental group. Indeed, while this increase may not have reached the stringent criteria for statistical significance, this difference may be clinically relevant. Additional studies are warranted to explore specific benefits to this degree of improvement in testosterone level. Moreover, given that serum DHT levels were significantly lower in both the 800 mg/day and 1200 mg/day treatment groups, and that Resettin®/MyTosterone™ has been shown to prevent the conversion of testosterone into DHT over time, it may be that this accounts for the rising testosterone levels in a subset of participants. Thus, additional studies that include a broader sample of study participants are warranted to explore for the generalizability of these findings. Future studies may also be needed to examine dosage level in relation to weight or BMI and androgen response. While weight specific dosing is not novel in terms of the pharmaceutical field, dietary supplements have not typically provided dosing instructions that are dependent upon the individual’s weight or BMI. It is expected that findings from studies examining the impact of various dosages of Resettin®/MyTosterone™ on the metabolic profiles, specifically testosterone, DHT, and estrogen levels, across individuals who are overweight or obese will provide support for including weight dependent dosing instructions and, thus, improve the individual’s hormonal response to this natural dietary supplement.

Additional studies are necessary to evaluate the full extent of the regulatory effects of Resettin® in the body’s efforts to resume homeostasis and return testosterone to ideal levels. This study highlights that there are likely ideal levels of testosterone in men. These data contribute to the possible benefits of using Resettin/Mytosterone for combating age-related androgen deficiency and andropause.

### Availability of supporting data

There is no supporting data that is currently available.

## Abbreviations

ANOVA-RM: Analysis of Variance-repeated measures

AX: Astaxanthin

DHT: Dihydrotestosterone

E2: Estradiol

ELISA: Enzyme-linked immunosorbent assay

HCG: Human chorionic gonadotropin

HRP: Horseradish peroxidase

SERMs: Selective estrogen receptor modulators

SHBG: Sex hormone binding globulin

## Competing interests

The author declares that he has no competing interests.

## Authors’ contributions

MA carried out experimental studies, participated in the randomized assignment of the participants and drafted the manuscript. MA carried out the immunoassays. MA participated in the design of the study and performed the statistical analysis. MA conceived of the study, and participated in its design and coordination and helped to draft the manuscript. The author has read and approved the final manuscript.
